# Rectal Urgency Among Patients With Ulcerative Colitis or Crohn’s Disease: Analyses from a Global Survey

**DOI:** 10.1093/crocol/otad052

**Published:** 2023-09-25

**Authors:** Christina Ha, April N Naegeli, Rina Lukanova, Mingyang Shan, Rosie Wild, Fritha Hennessy, Uma Jyothi Kommoju, Alison Potts Bleakman, Theresa Hunter Gibble

**Affiliations:** The F. Widjaja Foundation Inflammatory Bowel Immunology Research Institute at Cedars-Sinai, Los Angeles, CA, USA; Eli Lilly and Company, Indianapolis, IN, USA; Adelphi Real World, Bollington, Cheshire, UK; Eli Lilly and Company, Indianapolis, IN, USA; Adelphi Real World, Bollington, Cheshire, UK; Adelphi Real World, Bollington, Cheshire, UK; Eli Lilly and Company, Indianapolis, IN, USA; Eli Lilly and Company, Indianapolis, IN, USA; Eli Lilly and Company, Indianapolis, IN, USA

**Keywords:** rectal urgency, inflammatory bowel disease, ulcerative colitis, Crohn’s disease, patient-reported outcomes

## Abstract

**Background:**

Rectal urgency is a common but under-reported inflammatory bowel disease (IBD) symptom. The present study assessed the prevalence of rectal urgency and its association with disease activity and patient-reported outcomes (PROs) among patients with ulcerative colitis (UC) or Crohn’s disease (CD) in a real-world setting.

**Methods:**

Data were drawn from the 2017–2018 Adelphi IBD Disease Specific Programme™, a multi-center, point-in-time survey of gastroenterologists and consulting adult patients with UC or CD in France, Germany, Italy, Spain, the United Kingdom, and the United States. Gastroenterologists completed patient record forms and patients completed self-reported forms. Analyses were conducted separately for patients with UC or CD. Patient demographics, clinical characteristics, disease activity, symptoms, and PROs were compared between patients with and without rectal urgency.

**Results:**

In total, 1057 patients with UC and 1228 patients with CD were included. Rectal urgency was reported in 20.2% of patients with UC and 16.4% with CD. Patients with rectal urgency were more likely to have moderate or severe disease (UC or CD: *P* < .0001), higher mean Mayo score (UC: *P* < .0001), higher mean Crohn’s Disease Activity Index score (CD: *P* < .0001), lower Short IBD Questionnaire scores (UC or CD: *P* < .0001), and higher work impairment (UC: *P* < .0001; CD: *P* = .0001) than patients without rectal urgency.

**Conclusions:**

Rectal urgency is a common symptom associated with high disease activity, decreased work productivity, and worse quality of life. Further studies are needed to include rectal urgency assessment in routine clinical practice to better gauge disease activity in patients with UC or CD.

## Introduction

Ulcerative colitis (UC) and Crohn’s disease (CD) are inflammatory bowel diseases that cause chronic inflammation of the gastrointestinal tract.^1,2^ IBD imposes significant economic burden and negative impact on health-related quality of life (HRQoL) of patients.^2–4^ Rectal urgency, also referred to as bowel movement urgency or bowel urgency, is a sudden and immediate need to have a bowel movement and is a common symptom in patients with IBD.^5–10^ Rectal urgency is considered an important clinical manifestation and a strong predictor for clinical disease activity in UC or CD.^11,12^ Patients consider rectal urgency to be a more relevant and important symptom than abdominal pain, blood in stools, or stool frequency.^12,13^ While rectal urgency in UC is thought to be related to distal changes, in CD extensive disease is thought to cause symptoms of bowel dysfunction and may occur in the presence or absence of perianal disease.^14–17^ Even though rectal urgency has been associated with significant economic burden, morbidity, and impact on the patients' emotional, physiological, and social functioning, it is often under-reported in clinical practice and clinical trials.^6,9,10,15,18–22^ Limited global data are available on the prevalence of rectal urgency and its association with disease activity and patient-reported outcomes (PROs), such as EQ-5D-5L, Short IBD Questionnaire (SIBDQ), and Work Productivity and Activity Impairment (WPAI) among patients with UC or CD. The present study aimed to investigate the prevalence of rectal urgency in patients with UC or CD in a global cross-sectional survey. We also evaluated the association of rectal urgency with disease activity and PROs.

## Materials and Methods

### Data Source and Collection

Data were drawn from the Adelphi IBD Disease Specific Programme (DSP), a point-in-time survey.^23^ The Adelphi IBD DSP consists of data pertaining to treatment practice, symptom prevalence, patient demographics, clinical outcomes, medication utilization, healthcare utilization, productivity, and HRQoL.^23–25^ Gastroenterologists and their consulting adult patients with UC or CD from France, Germany, Italy, Spain, the United Kingdom, and the United States were surveyed. Data were collected via gastroenterologist-completed patient record forms (PRF) and patient-self-completed (PSC) forms. The terms “bowel urgency” and “rectal urgency” are generally used synonymously in the literature.^5–10^ Here, we used “rectal urgency” throughout the study as it was the term used in the survey conducted as a part of Adelphi DSP.

Gastroenterologists were identified from publicly available lists of healthcare professionals (HCPs) and invited to participate in the survey. The gastroenterologists had to have treated at least seven patients with UC and at least eight patients with CD on average in a month. Patients aged > 18 years with a diagnosis of UC or CD, as recorded on the PRF, were included. Patients were required to have a gastroenterology clinic visit during the study period. Gastroenterologists were requested to complete a PRF for the next seven to eight consecutive patients who visited them for consultation. The PRF contained detailed questions on patient demographics, clinical characteristics (disease severity and symptoms), disease activity, and medication use. Categories related to remission status (not in remission or in remission) were included and reported by the gastroenterologist. Following physician consultation, patients were invited to voluntarily complete a paper survey using PSC forms. All participating gastroenterologists and patients were assigned a study number to aid anonymous data collection and to allow linkage of data during data collection and analysis.

The PSC form contained questions on general symptoms including the following: (1) Which of the following symptoms they are currently experiencing: rectal urgency, fatigue, abdominal pain, sleep disturbance, etc.? (2) What is the current level of severity for each symptom?, and (3) Which are the top three symptoms most difficult to resolve? Levels of overall pain and sleep disturbance were measured on a scale of 0–10 (0 = no pain or no sleep disturbance to 10 = severe pain or sleep disturbance). The PSC form also contained PROs such as the WPAI, SIBDQ, and EQ-5D-5L. The WPAI: General Health V2.0 measures work productivity-related domains (absenteeism, presenteeism, work productivity loss, and activity impairment). Higher impairment percentages indicate greater work impairment and less productivity.^26^ The SIBDQ instrument measures various aspects of HRQoL (bowel, systemic, social, and emotional domains), with scores ranging from 10 to 70. Lower SIBDQ scores indicate poor HRQoL.^27,28^ The EQ-5D-5L, which is based on EQ-5D,^29,30^ measures health in five dimensions (mobility, self-care, usual activities, pain/discomfort, and anxiety/depression) using five levels (no problems, slight problems, moderate problems, severe problems, and extreme problems).^31^

### Outcome Measures

Patient demographics included age, sex, ethnic origin, smoking status, employment status, current (at point of consultation and data collection) level of disease severity (UC or CD), and current type of CD. Disease activity was measured by Mayo score (UC) and Crohn’s Disease Activity Index (CDAI) score (CD). Mayo score was calculated based on stool frequency, rectal bleeding, endoscopic findings, and Physician’s Global Assessment (PGA). Symptoms captured included level of abdominal pain and fatigue/tiredness. For CDAI, general well-being, extra-intestinal conditions, and presence of an abdominal mass were recorded. Severity of abdominal pain and current IBD medication regimen (biologics/non-biologics) were recorded for patients with UC or CD. Quality of life measures included EQ-5D-5L, SIBDQ, and WPAI. The results present in the study included combined survey results of both gastroenterologists and patients.

### Statistical Methods

Demographics, clinical characteristics, disease activity, symptoms, and PROs were compared between patients with and without rectal urgency using parametric and non-parametric tests, where appropriate. *T*-tests were performed to determine statistical differences between the means of the groups for numerical outcomes. Fisher’s exact test was used for dichotomous variables, and Chi-squared test was used for categorical variables. All analyses were conducted for UC or CD groups separately. Missing data were not imputed; therefore, the number of patients included for analysis may vary for each variable and is reported separately for each analysis.

Data were presented as mean and standard deviation (SD) for continuous variables. Frequencies and percentages were reported for categorical variables. Logistic regressions were used to identify risk factors associated with rectal urgency. In the regression models, rectal urgency was considered as the dependent variable. Independent variables included disease activity (Mayo for UC; CDAI for CD), fatigue, abdominal pain, overall pain, sleep disturbance, stool frequency (UC only), rectal bleeding (UC only), severity of abdominal pain (CD only), general well-being (CD only), PRO scales (EQ-5D-5L, SIBDQ, and WPAI), and medication use. Regressions were adjusted for confounders: age, sex, body mass index, and the Charlson Comorbidity Index. Standard errors from the regressions were adjusted to allow for intra-group correlation within reporting physician, relaxing the assumption that all observations are independent.

Odds ratios (ORs) with 95% confidence interval (CI) were reported for each of the covariates used in the regression model. Two-sided *P*-values were considered for all the statistical tests, and a significance level of *α* < .05 was considered to be statistically significant. All statistical tests were performed using Stata software (Version 16) (Stata Corp. 2019).

## Ethical Considerations

All the survey responses captured on the data collection forms were anonymized to preserve respondent (physician and patient) confidentiality. All materials were reviewed and approved by the Western Institutional Review Board. Patients provided their consent to complete the PSC form in accordance with the Health Insurance Portability and Accountability Act,^32^ Health Information Technology for Economic and Clinical Health Act (HITECH Act), and the European Pharmaceutical Market Research Association (EphMRA) Code of Conduct.^33,34^ The DSP fieldwork teams adhered to national data collection regulations (European Society for Opinion and Market Research [ESOMAR]^35^; US Department of Health and Human Services, National Institutes of Health, Health Insurance Portability and Accountability Act^32^; and The Market Research Society, British Standards Institute).^36^

## Results

### Study Population

A total of 380 gastroenterologists were included in the study (United States: *n* = 100, United Kingdom: *n* = 33, Germany: *n* = 60, France: *n* = 64, Italy: *n* = 62, Spain: *n* = 61) during 2017–2018. Overall, 2608 consulting patients with UC and 3003 patients with CD were recruited. Of these, 1057 patients with UC and 1228 patients with CD completed the PSC form and were included in the analysis. The patients included in the analysis were from France (UC: 208, CD: 255), Germany (UC: 303, CD: 350), Italy (UC: 76, CD: 101), Spain (UC: 160, CD: 150), United Kingdom (UC: 40, CD: 44), and the United States (UC: 270, CD: 328). The results pertain to global analyses and are not presented as region-specific data.

### Prevalence of Rectal Urgency and Patient Characteristics in the UC Cohort

A total of 20.2% (*n* = 213) of patients with UC reported rectal urgency (Table 1). Overall, 47% and 49% of patients were female in the rectal urgency and without rectal urgency groups, respectively. Mean age (SD) of patients with and without rectal urgency was 38.6 (14.8) years and 40.5 (14.9) years, respectively. A majority of the patients were White/Caucasian in both with and without rectal urgency groups (Table 1). Patients with rectal urgency presented a higher disease burden than those without in both moderate (54.5% vs. 38.5%) and severe categories of disease severity (8% vs. 4.5%), as assessed by the PGA. Demographic variables were not significantly different between patients with and without rectal urgency (Table 1).

**Table 1.** Comparison of demographics and clinical characteristics among patients with ulcerative colitis, with and without rectal urgency

	Rectal Urgency (*n* = 213)	No Rectal Urgency (*n* = 844)	*P*-value
Age (years), mean (SD)	38.6 (14.8)	40.5 (14.9)	.1031
Female, *n* (%)	100 (46.9)	414 (49.1)	.5922
Patient’s ethnic origin, *n* (%)			
White/Caucasian	189 (88.7)	743 (88.0)	.1435
African American	2 (0.9)	28 (3.3)	
Asian/Middle Eastern	5 (2.3)	30 (3.6)	
Hispanic/Latino	9 (4.2)	16 (1.9)	
Afro-Caribbean	3 (1.4)	11 (1.3)	
Mixed Race/Other	5 (2.3)	16 (1.9)	
Smoking status, *n* (%)	*n* = 197	*n* = 772	.0812
Current smoker	30 (15.2)	75 (9.7)	
Ex-smoker	53 (26.9)	213 (27.6)	
Never smoked	114 (57.9)	484 (62.7)	
Employment status, *n* (%)	*n* = 206	*n* = 823	.1468
Working full-time	118 (57.3)	480 (58.3)	
Working part time	15 (7.3)	74 (9.0)	
On long-term sick-leave	8 (3.9)	14 (1.7)	
Homemaker	9 (4.4)	65 (7.9)	
Student	27 (13.1)	81 (9.8)	
Retired	18 (8.7)	76 (9.2)	
Unemployed	11 (5.3)	33 (4.0)	
Disease severity of UC, *n* (%)			
Mild	75 (35.2)	464 (55.0)	<.0001*
Moderate	123 (57.7)	365 (43.2)	
Severe	15 (7.0)	15 (1.8)	
Current remission status, *n* (%)	*n* = 213	*n* = 844	
Not in remission	125 (58.7)	325 (38.5)	<.0001*
In remission	88 (41.3)	519 (61.5)	
Disease activity: Mayo score, mean (SD)	5.5 (2.6)	3.9 (2.9)	<.0001*
Endoscopic findings, *n* (%)			
Normal or inactive disease	23 (10.8)	265 (31.4)	<.0001*
Mild disease	67 (31.5)	261 (30.9)	
Moderate disease	107 (50.2)	281 (33.3)	
Severe disease	16 (7.5)	37 (4.4)	
PGA, *n* (%)			
Normal	11 (5.2)	198 (23.5)	<.0001*
Mild disease	69 (32.4)	283 (33.5)	
Moderate disease	116 (54.5)	325 (38.5)	
Severe disease	17 (8.0)	38 (4.5)	
Stool frequency, *n* (%)			
Normal # of stools for patient	30 (14.1)	292 (34.6)	<.0001*
1–2 stools per day more than normal	90 (42.3)	341 (40.4)	
3–4 stools more than normal	54 (25.4)	166 (19.7)	
5 or more stools more than normal	39 (18.3)	45 (5.3)	
Rectal bleeding, *n* (%)			
No blood seen	82 (38.5)	468 (55.5)	<.0001*
Streaks of blood in stool less than half the time	91 (42.7)	280 (33.2)	
Obvious blood with stool most of the time	39 (18.3)	90 (10.7)	
Blood alone passed	1 (0.5)	6 (0.7)	
Current symptoms			
Fatigue, *n* (%)	72 (33.8)	167 (19.8)	<.0001*
Abdominal pain, *n* (%)	75 (35.2)	176 (20.9)	<.0001*
Level of pain^a^, mean (SD)	3.9 (2.4)	2.7 (2.4)	<.0001*
Level of sleep disturbance^b^, mean (SD)	3.4 (2.4)	2.3 (2.3)	<.0001*
Quality of life			
EQ-5D-5L, mean (SD)	*n* = 210; 0.8^c^ (0.2)	*n* = 829; 0.8^c^ (0.2)	<.0001*
* *SIBDQ, mean (SD)	*n = 179; 4.6 (1.0)*	*n* = 714; 5.3 (1.1)	<.0001*
WPAI: Overall work impairment, mean (SD)	*n* = 102; 41.8 (30.1)	*n* = 372; 25.0 (26.9)	<.0001*
Current medication, *n* (%)			
5-ASA	176 (82.6)	739 (87.6)	.0714
Corticosteroid	164 (77.0)	601 (71.2)	.1032
Immunomodulator	108 (50.7)	416 (49.3)	.7591
Biologic	76 (35.7)	340 (40.3)	.2393
Time from initiation of current medication to data collection (weeks), mean (SD)			
5-ASA	*n* = 167; 38.9 (65.5)	*n* = 729; 63.3 (107.4)	.0049*
Corticosteroid	*n* = 154; 33.4 (38.3)	*n* = 592; 67.1 (108.9)	.0002*
Immunomodulator	*n* = 107; 36.4 (40.7)	*n* = 413; 64.9 (98.6)	.0037*
Biologic	*n* = 74; 44.0 (87.4)	*n* = 335; 63.3 (95.6)	.1120
Mode of administration of corticosteroids	*n* = 64	*n* = 120	
Enema	6 (9.4)	15 (12.5)	.6307
Tablets	47 (73.4)	79 (65.8)	.3211
Capsules	5 (7.8)	11 (9.2)	1.0000
Granules	1 (1.6)	2 (1.7)	1.0000
Foam	4 (6.3)	10 (8.3)	.7736
Other	3 (4.7)	4 (3.3)	.6956

*n* = number of patients; ASA = aminosalicylic acid; UC = ulcerative colitis; SD = standard deviation; SIBDQ = Short Inflammatory Bowel Disease Questionnaire; WPAI = Work Productivity and Activity Impairment; PGA = Physician’s Global Assessment; ^a^Level of pain was measured on a scale from 0 = no pain to 10 = severe pain; ^b^Level of sleep disturbance was measured on a scale from 0 = no sleep disturbance to 10 = severe sleep disturbance; ^c^Data are rounded off to single decimal points (Mean EQ-5D-5L scores: rectal urgency = .800 and no rectal urgency = .848); *Statistical significance of *α* < .05. T-test was conducted for numerical data; Fisher exact test for dichotomous outcomes; Chi-square test for categorical variables.

### Prevalence of Rectal Urgency and Patient Characteristics in the CD Cohort

A total of 16.4% (*n* = 202) of patients with CD reported rectal urgency (Table 2). Overall, 46.5% and 51.0% of patients were female in the rectal urgency and without rectal urgency groups, respectively. The mean (SD) age of patients with and without rectal urgency was 38.7 (13.1) and 38.5 (13.6) years, respectively. A majority of the patients were White/Caucasian in both with and without rectal urgency groups (Table 2). Patients with rectal urgency were overrepresented in the moderate disease category compared to those without (59.4% vs. 35.0%), whereas they were slightly underrepresented in the severe disease category (4.5% vs. 5.5%). Demographic variables were not significantly different between patients with and without rectal urgency (Table 2).

**Table 2.** Comparison of demographics and clinical characteristics among patients with Crohn’s disease, with and without rectal urgency

	Rectal Urgency (*n* = 202)	No Rectal Urgency (*n* = 1026)	*P*-value
Age (years), mean (SD)	38.7 (13.1)	38.5 (13.6)	.8452
Female, *n* (%)	94 (46.5)	523 (51.0)	.2812
Patient’s ethnic origin, *n* (%)			
White/Caucasian	186 (92.1)	915 (89.2)	.7318
African American	3 (1.5)	22 (2.1)	
Asian/Middle Eastern	4 (2.0)	37 (3.6)	
Hispanic/Latino	3 (1.5)	21 (2.0)	
Afro-Caribbean	2 (1.0)	16 (1.6)	
Mixed Race/Other	4 (2.0)	15 (1.5)	
Smoking status, *n* (%)	*n* = 195	*n* = 962	
Current smoker	30 (15.4)	159 (16.5)	.3572
Ex-smoker	46 (23.6)	268 (27.9)	
Never smoked	119 (61.0)	535 (55.6)	
Employment status, *n* (%)	*n* = 199	*n* = 997	
Working full-time	105 (52.8)	547 (54.9)	.7487
Working part-time	29 (14.6)	118 (11.8)	
On long-term sick-leave	7 (3.5)	30 (3.0)	
Homemaker	9 (4.5)	70 (7.0)	
Student	26 (13.1)	121 (12.1)	
Retired	14 (7.0)	59 (5.9)	
Unemployed	9 (4.5)	52 (5.2)	
Disease severity of CD, *n* (%)			
Mild	73 (36.1)	611 (59.6)	<.0001*
Moderate	120 (59.4)	359 (35.0)	
Severe	9 (4.5)	56 (5.5)	
Current remission status, *n* (%)	*n* = 202	*n* = 1026	
Not in remission	115 (56.9)	380 (37.0)	<.0001*
In remission (complete mucosal healing has occurred and CDAI < 150)	87 (43.1)	646 (63.0)	
Type of CD, *n* (%)			
Crohn’s ileitis	43 (21.3)	295 (28.8)	.0312*
Crohn’s colitis	73 (36.1)	288 (28.1)	.0228*
Crohn’s ileocolitis	82 (40.6)	363 (35.4)	.1735
Jejunoileitis	4 (2.0)	44 (4.3)	.1625
Gastroduodenal CD	9 (4.5)	21 (2.0)	.0750
Other	5 (2.5)	35 (3.4)	.6646
Current medication, *n* (%)			
5-ASA	147 (72.8)	664 (64.7)	.0282*
Corticosteroid	153 (75.7)	737 (71.8)	.2641
Immunomodulator	105 (52.0)	559 (54.5)	.5371
Biologic	77 (38.1)	493 (48.1)	.0108*
Time from initiation of current medication to data collection (weeks), mean (SD)			
5-ASA	*n* = 140; 47.2 (62.9)	*n* = 653; 62.2 (100.7)	.0920
Corticosteroid	*n* = 147; 45.6 (63.2)	*n* = 724; 57.2 (76.9)	.0859
Immunomodulator	*n* = 104; 60.6 (85.2)	*n* = 556; 62.8 (86.2)	.8078
Biologic	*n* = 74; 50.8 (93.6)	*n* = 491; 65.7 (87.7)	.1784
Mode of administration of corticosteroids	*n* = 48	*n* = 169	
Enema	2 (4.2)	8 (4.7)	1.0000
Tablets	28 (58.3)	109 (64.5)	.4984
Capsules	14 (29.2)	44 (26.0)	.7126
Granules	0 (0.0)	3 (1.8)	1.0000
Foam	2 (4.2)	4 (2.4)	.6159
Other	2 (4.2)	3 (1.8)	.3060
CDAI, mean (SD)	134.3 (74.9)	103.3 (78.5)	<.0001*
Average number of stools experienced per day, mean (SD)	*n* = 195 1.9 (1.7)	*n* = 892 1.5 (1.5)	.0008*
Average abdominal pain rating experience per day, mean (SD)	*n* = 197 1.2 (0.7)	*n* = 925 0.9 (0.8)	<.0001*
Current symptoms			
Fatigue, *n* (%)	74 (36.6)	246 (24.0)	.0003*
Abdominal pain, *n* (%)	95 (47.0)	341 (33.2)	.0003*
Level of pain^a^, mean (SD)	4.0 (1.9)	3.1 (2.5)	<.0001*
Level of sleep disturbance^a^, mean (SD)	3.1 (2.0)	2.5 (2.4)	.0007*
Quality of life			
EQ5D-5L, mean (SD)	*n* = 201; 0.8 (0.1)	*n* = 1007; 0.9 (0.1)	.0092*
SIBDQ, mean (SD)	*n* = 150; 4.7 (1.0)	*n* = 875; 5.2 (1.1)	<.0001*
WPAI: Overall work impairment, mean (SD)	*n* = 82; 35.6 (25.8)	*n* = 460; 24.0 (24.8)	.0001*

*n* = number of patients; ASA = aminosalicylic acid; CD = Crohn’s disease; CDAI = Crohn’s Disease Activity Index; SD = standard deviation; SIBDQ = Short Inflammatory Bowel Disease Questionnaire; WPAI = Work Productivity and Activity Impairment;

^a^Level of pain was measured on a scale from 0 = no pain to 10 = severe pain;

^b^Level of sleep disturbance was measured on a scale from 0 = no sleep disturbance to 10 = severe sleep disturbance; *statistical significance of *α* < .05.

### Association of Disease Activity and PROs With Rectal Urgency in the UC Cohort

Patients reporting rectal urgency were more likely to have moderate or severe disease (*P* < .0001) and a higher mean Mayo score (*P* < .0001) compared to those without rectal urgency (Table 1). Similarly, patients with rectal urgency were less likely to be in remission and have a disease activity index score of 0 compared to patients without rectal urgency (*P* < .0001). Patients with rectal urgency were more likely to have active disease, particularly moderate or severe disease, compared to patients without rectal urgency (*P* < .0001; Table 1).

**Figure 1.**
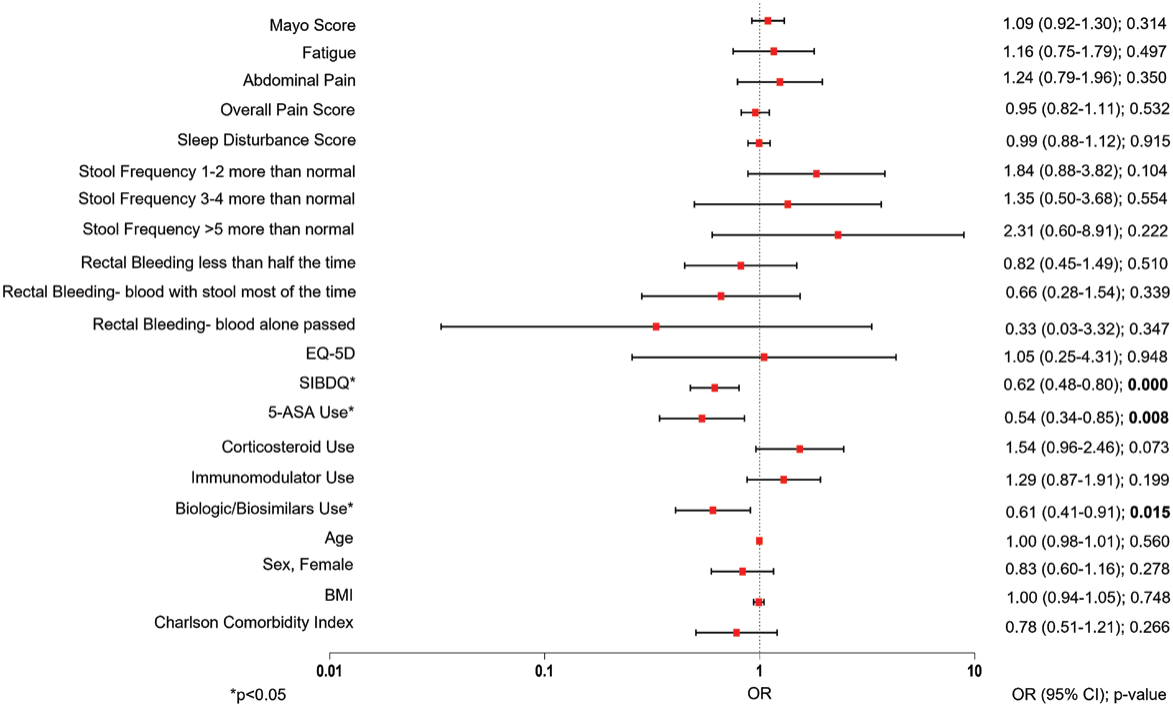
Logistic regression analyses for rectal urgency in patients with ulcerative colitis. ASA = aminosalicylic acid; BMI = body mass index; CI = confidence interval; SIBDQ = Short Inflammatory Bowel Disease Questionnaire; UC = ulcerative colitis; OR = odds ratio; Odds ratios were computed in the regressions and, for categorical outcomes, are in comparison to the baseline reference categories: No fatigue, no abdominal pain, stool frequency (normal number of stools), no blood seen, medication use (not 5-ASA, not corticosteroid, not immunomodulators, not biologics/biosimilars).

Patients with rectal urgency reported a greater degree of stool frequency and rectal bleeding than patients without rectal urgency (*P* < .0001; Table 1). Patients with rectal urgency were more likely to have fatigue (*P* < .0001), abdominal pain (*P* < .0001), higher levels of overall pain (*P* < .0001), and higher levels of sleep disturbance than patients without rectal urgency (*P* < .0001; Table 1). The mean SIBDQ and EQ-5D-5L scores were lower and the mean WPAI scores were higher in patients with rectal urgency compared to those without rectal urgency, indicating a lower HRQoL in patients with rectal urgency (SIBDQ mean [SD]: 4.6 [1.0] vs. 5.3 [1.1], *P* < .0001; EQ-5D-5L: 0.8 [0.2] vs. 0.8 [0.2], *P* < .0001; WPAI: 41.8 [30.1] vs. 25.0 [26.9], *P* < .0001; Table 1). Medication use was not significantly different in patients with and without rectal urgency (Table 1). Logistic regression analysis observed that the odds of having rectal urgency in patients with UC were lower with higher SIBDQ scores (OR: 0.62; 95% CI: [0.48 − 0.80]; *P* < .0001); 5-aminosalicylic acid (ASA) use (OR: 0.54; 95% CI: [0.34 − 0.85]; *P* = .008), and with the use of biologics/biosimilars (OR: 0.61; 95% CI: [0.41 − 0.91]; *P* = .015; Figure 1). Among patients with rectal urgency, stool frequency, and rectal bleeding were not significantly different when compared to patients without rectal urgency (Figure 1).

### Association of Disease Activity and PROs With Rectal Urgency in the CD Cohort

The current remission status was significantly different among CD patients with and without rectal urgency (*P* < .0001; Table 2). Patients with rectal urgency were more likely to have active disease (*P* < .0001), particularly moderate or severe disease, as well as higher mean CDAI scores (*P* < .0001) compared to patients without rectal urgency (Table 2). A higher percentage of patients with rectal urgency were prescribed 5-ASAs (72.8% vs. 64.7%; *P* = .0282), while biologics use was most prevalent amongst patients without rectal urgency (38.1% vs. 48.1%; *P* = .0108; Table 2).

**Figure 2.**
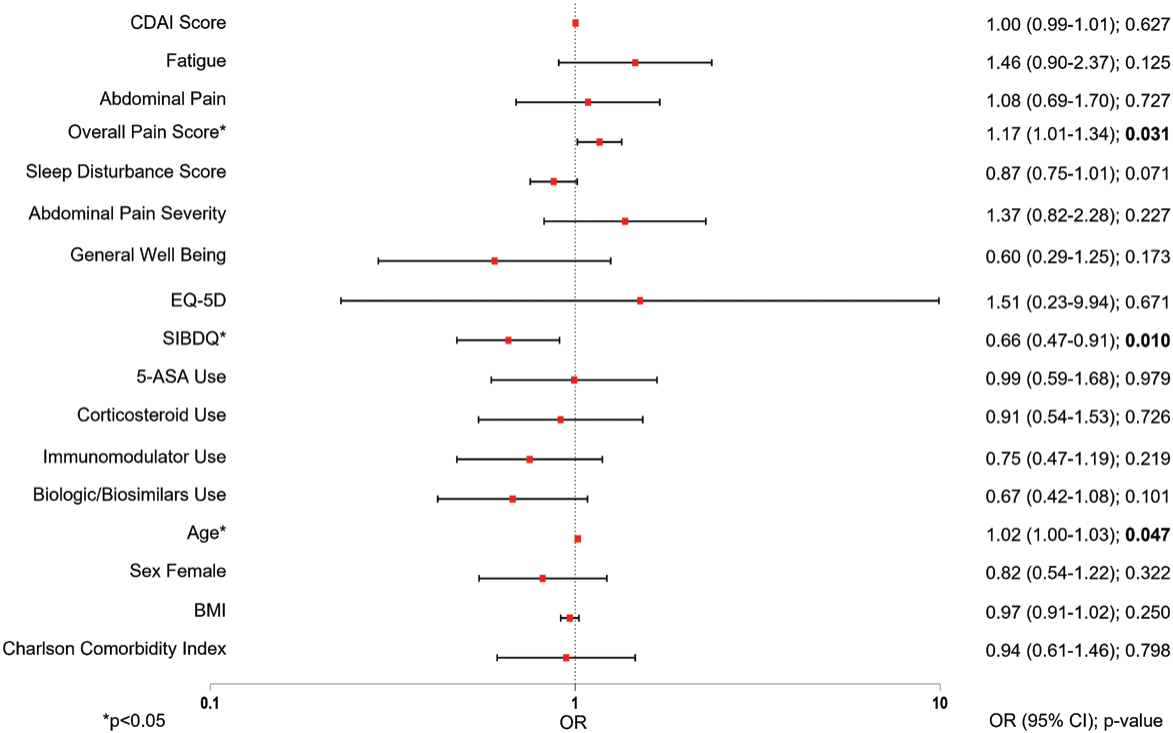
Logistic regression analyses for rectal urgency in patients with Crohn’s disease. ASA = aminosalicylic acid; BMI = body mass index; CD = Crohn’s disease; CDAI = Crohn’s Disease Activity Index; CI = confidence interval; SIBDQ = Short Inflammatory Bowel Disease Questionnaire; OR = odds ratio; Odds ratios were computed in the regressions and, for categorical outcomes, are in comparison to the baseline reference categories: No fatigue, no abdominal pain, stool frequency (normal number of stools), no blood seen, medication use (not 5-ASA, not corticosteroid, not immunomodulators, not biologics/biosimilars).

Patients with rectal urgency were more likely to have fatigue (*P* = .0003), abdominal pain (*P* = .0003), a higher level of pain (*P* < .0001), and a higher level of sleep disturbance than patients without rectal urgency (*P* = .0007; Table 2). Patients with rectal urgency also had lower SIBDQ (mean [SD]: 4.7 [1.0] vs. 5.2 [1.1], *P* < .0001) and EQ-5D-5L (0.8 [0.1] vs. 0.9 [0.1], *P* = .0092) scores and higher work impairment than patients without rectal urgency (35.6 [25.8] vs. 24.0 [24.8], *P* = .0001; Table 2). Logistic regression analyses showed that among patients with rectal urgency, the odds of having rectal urgency increased with higher overall pain score (OR: 1.17; 95% CI: [1.01 − 1.34]; *P* = .031) and decreased with higher SIBDQ scores (OR: 0.66; 95% CI: [0.47 − 0.91]; *P* = .010), 5-ASA use (OR: 0.99; 95% CI: [0.59 − 1.68]; *P* = .979), and use of biologics/biosimilars (OR: 0.67; 95% CI: [0.42 − 1.08]; *P* = .101; Figure 2).

## Discussion

Rectal urgency is an important symptom experienced by patients with IBD.^10^ It contributes to reduced quality of life; however, it remains under-investigated to date. The present study was conducted based on data drawn from a multinational point-in-time survey of gastroenterologists and their consulting patients with UC or CD to assess the burden of rectal urgency in a real-world clinical practice setting. In general, the terms “bowel urgency” and “rectal urgency” are used interchangeably in IBD studies.^5–10^ However, we used “rectal urgency” throughout the current study as it was the term used in the survey conducted as a part of Adelphi DSP.

In this study, the prevalence of rectal urgency was 20% among patients with UC and 16% among patients with CD. Our findings suggest that patients with UC or CD having rectal urgency were more likely to have moderate or severe disease compared to those without rectal urgency, while patients without rectal urgency were more likely to be in remission. Our findings also suggest that rectal urgency was significantly associated with higher disease activity (Mayo score in patients with UC; CDAI score in patients with CD) and lower SIBDQ (poorer HRQoL). However, the use of agents with known mucosal healing outcomes,^37^ such as biologics and 5-ASAs, was associated with decreased likelihood of rectal urgency. Similarly, increased overall pain scores and reduced SIBDQ scores were significantly associated with rectal urgency in patients with CD. These findings indicate reduced well-being among IBD patients with rectal urgency.

To date, only a few global studies have estimated rectal urgency in patients with UC or CD. Previous clinical studies reported the presence of rectal urgency in 31.4%–91.7% of patients with UC and 33.8%–86.7% of patients with CD.^4,7,9,10,15,18,38,39^ The difference in prevalence of rectal urgency between current and previous studies could be attributed to the differences in disease severity/remission status and lack of inclusion of definition of rectal urgency in the survey questions. In another real-world study, greater disease activity at enrollment in the cohort was associated with deteriorating fecal urgency symptoms over 6 months in patients with UC or CD.^40^

Even though our data show the meaningful impact of rectal urgency on IBD patients’ clinical activity and quality of life, rectal urgency is often not discussed in clinical practice due to patient hesitation or embarrassment.^15,19^ Rectal urgency may not be adequately addressed by HCPs due to lack of awareness regarding its importance as a clinical symptom, since it is not part of commonly used disease activity metrics such as the Mayo score or the PRO-2.^6,9,41–43^ Validated PROs developed per FDA guidance (FDA 2009) to assess bowel urgency,^44^ such as the Urgency Numeric Rating Scale (Urgency NRS),^41^ have only recently become available. Rectal urgency is not only an important marker of disease activity, but also a key factor that should be considered when assessing response to therapy and remission. Future research should include rectal urgency assessments when developing robust and reliable clinical indices for clinical practice and clinical trials. HCPs should include rectal urgency in their routine assessments when evaluating patients with IBD and factor the presence of urgency into shared decision-making and treat to target discussions.

### Limitations

Given the nature of the point-in-time study design, there may be recall bias as patients had to fill in PSC forms. However, the data for these analyses were collected at the time of each patient’s appointment, and this is expected to reduce the likelihood of recall bias. As the recruitment strategy focused only on gastroenterologist consulting patients, the results cannot be generalized.

## Conclusions

In the current study, rectal urgency was identified as a common symptom among patients with UC or CD and was associated with higher levels of disease activity, decreased work productivity, and worse HRQoL. Rectal urgency is an important PRO to consider when monitoring response to treatment in addition to stool frequency or rectal bleeding for patients with UC and stool frequency or abdominal pain for patients with CD. Therefore, a better understanding of the PROs and clinical manifestations associated with rectal urgency in patients with UC or CD may help improve disease control. Further investigation may be required to standardize rectal urgency assessment as a metric of disease activity in clinical practice.

## Data Availability

All data that support the findings of this study are the intellectual property of a third-party vendor. All requests for access should be addressed directly to the point of contact of the third-party vendor.

## References

[1] Kaistha A , Levine J. Inflammatory bowel disease: the classic gastrointestinal autoimmune disease. Curr Probl Pediatr Adolesc Health Care. 2014;44(11):328-334. 25499459 10.1016/j.cppeds.2014.10.003

[2] Le Berre C , Ananthakrishnan AN, Danese S, Singh S, Peyrin-Biroulet L. Ulcerative colitis and Crohn’s disease have similar burden and goals for treatment. Clin Gastroenterol Hepatol. 2020;18(1):14-23. 31301452 10.1016/j.cgh.2019.07.005

[3] Perera S , Yang S, Stott-Miller M, Brady J. Analysis of healthcare resource utilization and costs after the initiation of biologic treatment in patients with ulcerative colitis and Crohn’s disease. J Health Econ Outcomes Res. 2018;6(1):96-112. 32685575 10.36469/9791 PMC7309948

[4] Rubin DT , Sninsky C, Siegmund B, et al. International perspectives on management of inflammatory bowel disease: opinion differences and similarities between patients and physicians from the IBD GAPPS survey. Inflamm Bowel Dis. 2021;27(12):1942-1953. 33512475 10.1093/ibd/izab006 PMC8637792

[5] Ha C , Naegeli AN, Lukanova R, Shan M, Hunter T. Sa070 rectal urgency among ulcerative colitis and Crohn’s disease patients: analyses from a global survey. Gastroenterology. 2021;160(6):S-407-S-408.

[6] Sninsky JA , Barnes EL, Zhang X, Long MD. Urgency and its association with quality of life and clinical outcomes in patients with ulcerative colitis. Am J Gastroenterol. 2022;117(5):769-776. 35169109 10.14309/ajg.0000000000001685 PMC9064909

[7] Newton L , Randall JA, Hunter T, et al. A qualitative study exploring the health-related quality of life and symptomatic experiences of adults and adolescents with ulcerative colitis. J Patient Rep Outcomes. 2019;3(1):1-13. 31667633 10.1186/s41687-019-0154-x PMC6821900

[8] Papathanasopoulos A , Van Oudenhove L, Katsanos K, Christodoulou D, Tack J, Tsianos EV. Severity of fecal urgency and incontinence in inflammatory bowel disease: clinical, manometric and sonographic predictors. Inflamm Bowel Dis. 2013;19(11):2450-2456. 23949621 10.1097/MIB.0b013e3182a2952b

[9] Teich N , Schulze H, Knop J, Obermeier M, Stallmach A. Novel approaches identifying relevant patient-reported outcomes in patients with inflammatory bowel diseases—LISTEN 1. Crohns Colitis 360. 2021;3(3):otab050. 36776662 10.1093/crocol/otab050 PMC9802460

[10] Dawwas GK , Jajeh H, Shan M, Naegeli AN, Hunter T, Lewis JD. Prevalence and factors associated with fecal urgency among patients with ulcerative colitis and crohn’s disease in the study of a prospective adult research cohort with inflammatory bowel disease. Crohns Colitis 360. 2021;3(3):otab046. 36776663 10.1093/crocol/otab046 PMC9902261

[11] Van Deen WK , van der Meulen-de AE, Parekh NK, et al. Development and validation of an inflammatory bowel diseases monitoring index for use with mobile health technologies. Clin Gastroenterol Hepatol. 2016;14(12):1742-1750. e7. 26598228 10.1016/j.cgh.2015.10.035

[12] Van Deen WK , Obremskey A, Moore G, Van den Akker-van Marle M, Doctor JN, Hwang C. An assessment of symptom burden in inflammatory bowel diseases to develop a patient preference-weighted symptom score. Qual Life Res. 2020;29(12):3387-3396. 32813264 10.1007/s11136-020-02606-2

[13] Louis E , Ramos-Goñi JM, Cuervo J, et al. A qualitative research for defining meaningful attributes for the treatment of inflammatory bowel disease from the patient perspective. Patient. 2020;13(3):317-325. 31997116 10.1007/s40271-019-00407-5 PMC7210247

[14] Buchmann P , Kolb E, Alexander-Williams J. Pathogenesis of urgency in defaecation in Crohn’s disease. Digestion. 1981;22(6):310-316. 7333418 10.1159/000198676

[15] Petryszyn PW , Paradowski L. Stool patterns and symptoms of disordered anorectal function in patients with inflammatory bowel diseases. Adv Clin Exp Med. 2018;27(6):813-818. 29893516 10.17219/acem/68986

[16] Varbobitis I , Kokkotis G, Gizis M, et al. The IBD-F patient self-assessment scale accurately depicts the level of fatigue and predicts a negative effect on the quality of life of patients with IBD in clinical remission. Inflamm Bowel Dis. 2021;27(6):826-835. 32766770 10.1093/ibd/izaa201

[17] Mueller M , Kreis M, Gross M, Becker H, Zittel T, Jehle E. Anorectal functional disorders in the absence of anorectal inflammation in patients with Crohn’s disease. Br J Surg. 2002;89(8):1027-1031. 12153630 10.1046/j.1365-2168.2002.02173.x

[18] Nóbrega VG , Silva INN, Brito BS, Silva J, Silva MCM, Santana GO. The onset of clinical manifestations in inflammatory bowel disease patients. Arq Gastroenterol. 2018;55(3):290-295. 30540094 10.1590/S0004-2803.201800000-73

[19] Hibi T , Ishibashi T, Ikenoue Y, Yoshihara R, Nihei A, Kobayashi T. Ulcerative colitis: disease burden, impact on daily life, and reluctance to consult medical professionals: results from a Japanese internet survey. Inflamm Intest Dis. 2020;5(1):27-35. 32232052 10.1159/000505092 PMC7098303

[20] Gu P , Kuenzig ME, Kaplan GG, Pimentel M, Rezaie A. Fecal incontinence in inflammatory bowel disease: a systematic review and meta-analysis. Inflamm Bowel Dis. 2018;24(6):1280-1290. 29617820 10.1093/ibd/izx109

[21] Singh P , Takazawa E, Rangan V, et al. Fecal urgency is common in constipated patients and is associated with anxiety. Neurogastroenterol Motil. 2019;31(4):e13545. 30714267 10.1111/nmo.13545 PMC6414071

[22] Rangan V , Mitsuhashi S, Singh P, et al. Risk factors for fecal urgency among individuals with and without diarrhea, based on data from the National Health and Nutrition Examination Survey. Clin Gastroenterol Hepatol. 2018;16(9):1450-1458.e2. 29474972 10.1016/j.cgh.2018.02.020 PMC6098738

[23] Anderson P , Benford M, Harris N, Karavali M, Piercy J. Real-world physician and patient behaviour across countries: disease-specific programmes–a means to understand. Curr Med Res Opin. 2008;24(11):3063-3072. 18826746 10.1185/03007990802457040

[24] Babineaux SM , Curtis B, Holbrook T, Milligan G, Piercy J. Evidence for validity of a national physician and patient-reported, cross-sectional survey in China and UK: the Disease Specific Programme. BMJ Open. 2016;6(8):e010352. doi: 10.1136/bmjopen-2015-010352 PMC5013497 27531722

[25] Higgins V , Piercy J, Roughley A, et al. Trends in medication use in patients with type 2 diabetes mellitus: a long-term view of real-world treatment between 2000 and 2015. Diabetes Metab Syndr Obes. 2016;9:371-380. doi: 10.2147/dmso.S120101 27843332 PMC5098530

[26] Reilly MC , Zbrozek AS, Dukes EM. The validity and reproducibility of a work productivity and activity impairment instrument. PharmacoEcon. 1993;4(5):353-365. 10.2165/00019053-199304050-00006 10146874

[27] Irvine E , Zhou Q, Thompson A. The short inflammatory bowel disease questionnaire: a quality of life instrument for community physicians managing inflammatory bowel disease. Am J Gastroenterol. 1996;91(8):1571-1578. 8759664

[28] Jowett SL , Seal CJ, Barton JR, Welfare MR. The short inflammatory bowel disease questionnaire is reliable and responsive to clinically important change in ulcerative colitis. Am J Gastroenterol. 2001;96(10):2921-2928. 11693327 10.1111/j.1572-0241.2001.04682.x

[29] Brooks RE. EuroQoL: the current state of play. Health Policy. 1996;37(1):53-72. doi: 10.1016/0168-8510(96)00822-6 10158943

[30] EuroQol group, EuroQol--a new facility for the measurement of health-related quality of life. Health Policy. 1990;16(3):199-208. doi: 10.1016/0168-8510(90)90421-9 10109801

[31] Herdman M , Gudex C, Lloyd A, et al. Development and preliminary testing of the new five-level version of EQ-5D (EQ-5D-5L). Qual Life Res. 2011;20(10):1727-1736. 21479777 10.1007/s11136-011-9903-x PMC3220807

[32] US Department of Health and Human Services. OCR Privacy Brief Summary of the HIPAA Privacy Rule. Accessed August 9, 2022. http://www.hhs.gov/sites/default/files/privacysummary.pdf

[33] European Pharmaceutical Market Research Association (EphMRA). Code of Conduct. 2021. Accessed August 9, 2022. https://www.ephmra.org/code-conduct-aer

[34] Health Information Technology for Economic and Clinical Health Act (HITECH Act). Accessed August 9, 2022. https://www.healthit.gov/sites/default/files/hitech_act_excerpt_from_arra_with_index.pdf

[35] European Society for Opinion and Marketing Research (ESOMAR), International Code of Marketing and Social Research Practice. Accessed August 9, 2022. https://iccwbo.org/content/uploads/sites/3/2008/01/ESOMAR-INTERNATIONAL-CODE-ON-MARKET-AND-SOCIAL-RESEARCH.pdf

[36] Market Research Society, Market Research Society (MRS) Data Protection Act 1998 & Market Research: Guidance for MRS Members. Accessed August 9, 2022. https://www.mrs.org.uk/pdf/The%20Data%20Protection%20Act%201998%20and%20Market%20Research.pdf

[37] Atreya R , Neurath MF. Current and future targets for mucosal healing in inflammatory bowel disease. Visc Med. 2017;33(1):82-88. doi: 10.1159/000458006 28612022 PMC5465787

[38] Dulai PS , Jairath V, Khanna R, et al. Development of the symptoms and impacts questionnaire for Crohn’s disease and ulcerative colitis. Aliment Pharmacol Ther. 2020;51(11):1047-1066. 32319120 10.1111/apt.15726 PMC7317756

[39] Perler BK , Ungaro R, Baird G, et al. Presenting symptoms in inflammatory bowel disease: descriptive analysis of a community-based inception cohort. BMC Gastroenterol. 2019;19(1):1-8. 30940072 10.1186/s12876-019-0963-7 PMC6446285

[40] Naegeli A , Dong Y, Zhou X, Morris N, Arora V, Lissoos T. P121 Real world prevalence of bowel movement urgency–a snapshot of symptom experience reported by patients with ulcerative colitis participating in SPARC IBD. Inflamm Bowel Dis. 2020;26(suppl_1):S44-S45.

[41] Dubinsky MC , Irving PM, Panaccione R, et al. Incorporating patient experience into drug development for ulcerative colitis: development of the Urgency Numeric Rating Scale, a patient-reported outcome measure to assess bowel urgency in adults. J Patient Rep Outcomes. 2022;6(1):1-11. 35362902 10.1186/s41687-022-00439-w PMC8975984

[42] Dubinsky MC , Naegeli A, Dong Y, Lissoos T, Arora V, Irving P. P126 The Urgency Numeric Rating Scale (NRS): a novel patient-reported outcome measure to assess bowel urgency in adult patients with ulcerative colitis. J Crohns Colitis. 2020;14(suppl_1):S200-S200. doi: 10.1093/ecco-jcc/jjz203.255

[43] Higgins PD , Harding G, Revicki DA, et al. Development and validation of the Ulcerative Colitis patient-reported outcomes signs and symptoms (UC-pro/SS) diary. J Patient Rep Outcomes. 2018;2(1):1-9. 29888745 10.1186/s41687-018-0049-2 PMC5976680

[44] United States Food and Drug Administration. Patient-reported outcome measures: use in medical product development to support labeling claims: guidance for industry. 2009. Accessed August 9, 2022. https://www.fda.gov/media/77832/download 10.1186/1477-7525-4-79 PMC1629006 17034633

